# Knowledge in the Investigation of A-to-I RNA Editing Signals

**DOI:** 10.3389/fbioe.2015.00018

**Published:** 2015-02-24

**Authors:** Giovanni Nigita, Salvatore Alaimo, Alfredo Ferro, Rosalba Giugno, Alfredo Pulvirenti

**Affiliations:** ^1^Department of Molecular Virology, Immunology and Medical Genetics, Ohio State University, Columbus, OH, USA; ^2^Department of Mathematics and Computer Science, University of Catania, Catania, Italy; ^3^Department of Clinical and Experimental Medicine, University of Catania, Catania, Italy

**Keywords:** A-to-I RNA editing, motif analysis, prediction, ADARs, logistic regression

## Abstract

RNA editing is a post-transcriptional alteration of RNA sequences that is able to affect protein structure as well as RNA and protein expression. Adenosine-to-inosine (A-to-I) RNA editing is the most frequent and common post-transcriptional modification in human, where adenosine (A) deamination produces its conversion into inosine (I), which in turn is interpreted by the translation and splicing machineries as guanosine (G). The disruption of the editing machinery has been associated to various human diseases such as cancer or neurodegenerative diseases. This biological phenomenon is catalyzed by members of the adenosine deaminase acting on RNA (ADAR) family of enzymes and occurs on dsRNA structures. Despite the enormous efforts made in the last decade, the real biological function underlying such a phenomenon, as well as ADAR’s substrate features still remain unknown. In this work, we summarize the major computational aspects of predicting and understanding RNA editing events. We also investigate the detection of short motif sequences potentially characterizing RNA editing signals and the use of a logistic regression technique to model a predictor of RNA editing events. The latter, named AIRlINER, an algorithmic approach to assessment of A-to-I RNA editing sites in non-repetitive regions, is available as a web app at: http://alpha.dmi.unict.it/airliner/. Results and comparisons with the existing methods encourage our findings on both aspects.

## Background

In recent times, there has been a change in the range of research on many types of diseases. In the past decades, the principal aim was to add information about the molecular pathways involved in some disease through the study of DNA mutations. Lately, the focus has indeed moved to the analysis of post-transcriptional modification events, such as RNA editing. The knowledge that the activity of RNA editing is higher in mammalian brain than in other tissues (Paul and Bass, 1998), hints that editing may play a crucial role in the central nervous system (Nishikura, 2006). Therefore, malfunctions of RNA editing machineries could lead to serious consequences (Galeano et al., 2012; Tomaselli et al., 2014).

RNA editing is a type of post-transcriptional modification, taking place in eukaryotes, which alters the sequence of primary RNA transcripts by deleting, inserting, or modifying residues. Despite the discovery of several distinct types of RNA editing over the years, adenosine-to-inosine (A-to-I) RNA editing is now considered the most predominant in mammalians (Nishikura, 2010). Through the deamination process, adenosine (A) is converted into inosine (I), which in turn is interpreted as guanosine (G) by both the splicing and the translation machineries (Rueter et al., 1999). Enzymes members of the adenosine deaminase acting on RNA (ADAR) family catalyze this biological phenomenon only on dsRNA structures (Bass, 2002; Jepson and Reenan, 2008; Nishikura, 2010).

Adenosine-to-inosine RNA sites abundantly occur in intronic regions as well as in 3′-UTRs. RNA editing events can modify RNA molecules in several cellular contexts causing: the creation and/or destruction of splicing sites (Rueter et al., 1999); the modulation of gene expression pathways (Bazak et al., 2014b) during translation (Nishikura, 2010); the gain or loss of miRNA recognition elements (MRE) during mRNA targeting (Nishikura, 2006; Borchert et al., 2009) (i.e., MRE can be created or deleted even with a single post-transcriptional modification). As it has been reported in the last few years, RNA editing sites can be found in non-coding RNA molecules, especially within pri-miRNA (Kawahara et al., 2008; Kawahara, 2012), lncRNA (Mitra et al., 2012), and precursor-tRNA (Su and Randau, 2011), the latter deaminated by adenosine deaminases acting on tRNA (ADAT) enzymes.

It is possible to distinguish two forms of A-to-I RNA editing, *promiscuous* and *specific*. The *promiscuous* A-to-I editing occurs within longer duplexes of hundreds of nucleotides, as in the case of stem–loops that are formed by the pairing of repetitive elements (e.g., Alu elements), as seen above. In those cases, up to 60% of adenosines could be edited (Carmi et al., 2011; Bazak et al., 2014b). The *specific* A-to-I RNA editing occurs in short and/or unstable duplex RNA regions (Wahlstedt and O’Hman, 2011), in which at least 10% of their adenosines selectively could undergo deamination. A-to-I RNA editing events in small non-coding RNAs, such as microRNAs, are perfect examples of *specific* editing (Nishikura, 2010).

One of the main challenges in the study of the RNA editing phenomenon is certainly RNA editing occurrence. The detection of editing sites in RNA molecules in particular cellular conditions is very difficult considering that RNA editing is a dynamic spatial–temporal process. In the last decade, the application of global approaches to the study of A-to-I editing, including in a first phase bioinformatics methods and, lately, high-throughput sequencing technology (HTS) based pipelines, have led to important advances, allowing the discovery of a large amount of editing sites in the human transcriptome. Despite the enormous efforts made in recent years, the real biological function underlying such a phenomenon, as well as ADAR’s substrate features still remain unknown.

In this work, we give an overview of the current state of knowledge on the editing phenomenon, as well as provide the main features of editing sites as highlighted today. We also investigate, inspired by previous results, methods for the detection of signals characterizing editing events and the prediction of novel A-to-I editing sites in non-repetitive regions. These techniques are based on the analysis of nucleotide profiles within a distance-radius of the probable editing site. Results on the signal detection show that editing sites may not have strong defined signal patterns.

Finally, by using a logistic regression technique we developed AIRlINER, an algorithmic approach for the prediction of A-to-I RNA editing sites in non-repetitive regions. This method has been compared with *InosinePredict* (Eggington et al., 2011), a similar technique, which analyzes the nucleotides flanking the editing site. *InosinePredict* assumes a multiplicative relationship between the coefficients necessary to compute the percentage of editing. Our results clearly show that AIRlINER improves the quality of predictions with respect to *InosinePredict* and suggest further research directions. AIRlINER is available at the following address: http://alpha.dmi.unict.it/airliner/.

## Knowledge and Features of Editing Sites Signals

At the end of 80s, ADARs, initially identified as associated with an unknown dsRNA-unwinding activity (Bass and Weintraub, 1987; Rebagliati and Melton, 1987), were discovered as RNA editing machineries able to alter adenosine into inosine through deamination, especially in dsRNA structures (Bass and Weintraub, 1988; Wagner et al., 1989). In the next 10 years, three members of the ADAR gene family were identified in humans: two isoforms of ADAR1 (N-terminally truncated ADAR1p110 and a full-length ADAR1p150) (Kim et al., 1994; Patterson and Samuel, 1995), ADAR2 (Lai et al., 1997) (both these members expressed in many tissues), and ADAR3 (Chen et al., 2000) present only in the central nervous system. While for ADAR1 and ADAR2 the enzymatic activity was established, for ADAR3 it remains unknown. Unlike ADAR1 and ADAR2, an interesting feature about ADAR3 is the presence of the R domain, which enables the enzyme to bind to single strand structures. ADAR1 and ADAR2 have two common functional regions, an N-terminal dsRNA-binding domain (dsRBD) and a C-terminal deaminase domain, but only ADAR1 contains two Z-DNA-binding domains, Zα and Zβ. Some editing events are edited only by ADAR1 or ADAR2, showing a significant difference in their RNA-substrate interactions (Wong et al., 2001; Riedmann et al., 2008). For instance, the serotonin B site is deaminated not only by ADAR1, while the serotonin D and the GluR-B Q/R sites are deaminated exclusively by ADAR2 (Burns et al., 1997; Yang et al., 1997), but also ADAR1 and ADAR2 can edited the same target, as in the cases of serotonin A and C editing sites (Burns et al., 1997). Subsequently, the characterization of the neighborhood profiles of both ADAR1 and ADAR2 were established. In particular, ADAR1 has 5′ neighboring base preference consisting of uracil, adenosine, cytosine, and guanosine in order (U ≈ A > G > C), but not 3′neighbor preference has been identified (Polson and Bass, 1994). Similarly, ADAR2 has a 5′ neighbor preference, but, differently from ADAR1, ADAR2 has a 3′ neighboring base preference (U = G > C = A) forming particular trinucleotide sequences with an adenosine at the second base (U*A*U, A*A*G, U*A*G, A*A*U) (Lehmann and Bass, 2000).

In 2003, Hoopengardner et al. (2003) discovered that highly conserved regions, which in turn form a dsRNA structure, surround many editing sites. Later, by considering these findings, bioinformatics methods mapping ESTs against a reference genome were able to discover tens of thousands of A-to-I RNA editing sites, with more than 90% of them occurring within Alu repeats (Athanasiadis et al., 2004; Kim et al., 2004; Levanon et al., 2004). A significant problem in all the bioinformatics approaches for RNA editing detection, as described above, still remains the limitations posed by sequencing technologies, specifically, the inability to distinguish a guanosine originating from an I-to-G replacement from a guanosine as a product of noise, sequencing errors or SNP. A solution to this issue was proposed by Sakurai et al. (2010) who designed a biochemical method, called inosine chemical erasing (ICE), able to identify inosine sites on RNA molecules by employing inosine-specific cyanoethylation with reverse transcription. This is a reliable and accurate biochemical method to detect inosines in RNA strands.

The recent years have been characterized by the development of several approaches for editing discovery based on deep sequencing. It was recently hypothesized that more than 100 million editing sites could be found in human Alu repeats, located mainly in genic regions (Bazak et al., 2014a). Although these recent methods prove to be more accurate than previous ones, some of them nonetheless present limitations in terms of false positives produced (Kleinman and Majewski, 2012; Lin et al., 2012; Pickrell et al., 2012). In recent years, a considerable number of RNAseq based methods have emerged (Li et al., 2009; Ju et al., 2011; Bahn et al., 2012; Peng et al., 2012; Picardi et al., 2012; Ramaswami et al., 2012, 2013; Bazak et al., 2014a), gradually improved the accuracy in discovering new editing sites, leading, in addition, to the identification of a set of human editing sites orders of magnitude larger than before. Recently, Sakurai et al. (2014) combined the ICE method with HTS (ICE seq) for an unbiased genome-wide screening of novel A-to-I editing sites. ICE seq is able to detect editing sites in both repeat elements and short hairpins, rendering this a currently unique method for genome-wide identification of A-to-I editing events in both tissues and clinical specimens without genomic DNAs.

The application of HTS technology to RNA editing discovery has not only brought improvements in the editing discovery but also helped to increase the knowledge about the features inherent to the phenomenon. In fact, thanks to the analysis of a large RNA-seq data, Bazak et al. (2014b) studied the global characteristics that affect the editability at the Alu level, uncovering some important features. An important parameter that influences the editing of the Alu is the distance to the nearest complementary inverse sequence. Indeed, the editing, on average, exponentially decays with this distance, with a typical length of about 800 nt. Another aspect is that the editing levels are positively correlated with the number of reversely complementary repeats in the flanking regions of the Alu. Instead, they are negatively correlated with the number of same-strand repeats. Furthermore, the editing level depends on both the lengths of the Alu repeats and their closest reversely oriented sequence, additionally to whether the latter resides in the same intron/exon. Finally, the consensus strand of the Alus is more edited than the reverse strand.

Lately, Pinto et al. (2014) conducted a study with the scope to find mammalian conserved editing sites. Surprisingly, only a very small fraction (0.004%) of human editing sites is conserved in mammals. Noteworthy, by considering the nucleotide frequency, the 10-nt upstream and downstream regions of conserved editing sites are stronger than the ones of all non-Alu human editing sites.

The large number of editing sites discovered by these methodologies has given rise to the need for public databases to record such information in order to further elucidate the biological functions underlying the RNA editing phenomenon. The first centralized repository was DARNED^1^ (Kiran and Baranov, 2010), whose last release contains more than 300,000 editing sites (Kiran and Baranov, 2010; Kiran et al., 2013). Later, Ramaswami and Li (2014) built RADAR^2^, a rigorously manually curated database of annotated A-to-I editing sites, amounting to about 1.4 million editing events. Unfortunately, both DARNED and RADAR do not offer a grade of confidence for each editing site due to the heterogeneity of the discovering methods applied, making the creation of a standard measure of confidence necessary in the future.

## Investigation of Motifs Characterizing the RNA Editing Events

It is well known that the vast majority of editing events occur in repetitive regions. Recently, Ramaswami et al. (2012) developed a computational framework to identify editing events both Alu and non-Alu regions (repetitive non-Alu and non-repetitive regions) by analyzing the genomic DNA and RNA sequences. Through this method they found that more than 97% of the discovered editing events occur in Alu regions, also speculating that the remaining non-Alu editing sites are related to nearby edited Alu ones. This makes the identification of sequence motifs able to characterize RNA editing a very challenging problem. Therefore, any approach aimed at the search of sequence or structural motifs associated to RNA editing events should take into account the bias introduced by repetitive regions. Consequently, the searching should be done outside of repetitive regions in order to detect signals independent of the background.

Our strategy has been the following. First, we selected a set of non-Alu editing events and then generated edited regions (ERs) based on the distances between non-Alu editing site, as described below. Next, we applied MEME (Bailey et al., 2009) in order to discover motifs within such a set of sequences. MEME analyzes the input data and searches for significant ungapped sequence patterns shared among the sequences.

In order to obtain the ERs, considering the human editing sites listed in the RADAR database (Ramaswami and Li, 2014), we firstly filtered the A-to-I editing sites, which resulted to be SNPs, as compared to dbSNP141 (Solomon et al., 2014). We then computed δ as the weighted average distance between the editing sites. We obtained that on average there are 6,057 nt between two editing events. This value has been considered as a *breakpoint* during the construction of ERs. In particular, starting from a generic editing site *x*, we searched for the next one *y*. When *y* falls within a distance less than or equal to δ, the editing site *y* is included in the ER and the process continues. Otherwise, if the next site is found at a distance greater than δ, the ER is no longer extended. As a result, a total of 55,952 ERs have been defined. Additionally, we separated ERs containing repetitive elements from those, which do not contain any, obtaining a total of 48,164 repetitive ERs and 7,788 non-repetitive ERs. The fact that ERs possess different lengths could allow us to take into account the possibility that they may contain motifs close to the editing sites in secondary structures.

**Figure 1:**
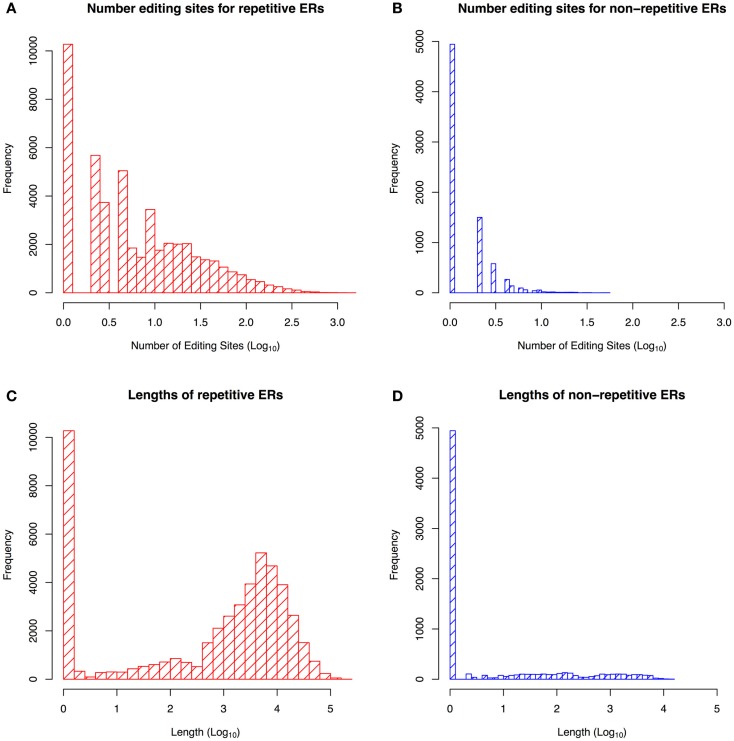
**Statistics about the *repetitive* and *non-repetitive* edited regions (ER)**. Distribution of editing sites frequency in *repetitive* ERs **(A)** and *non-repetitive* ERs **(B)**. Distribution of *repetitive* ERs sequence length **(C)** and *non-repetitive* ERs sequence length **(D)**. The figure shows that the non-repetitive ERs are shorter than repetitive ones and contain fewer editing sites.

Figure 1 shows that repetitive ERs are longer than non-repetitive ones, with the largest number of editing sites found in regions containing some repetitive elements, as confirmed in the literature (Wahlstedt and O’Hman, 2011). We built a training set of non-repetitive ERs by selecting those regions with a length of 2,000–6,000 nt, containing at least 10 editing sites. Hence, we obtained a final dataset of 47 ERs, in particular, 29 regions are in positive strand with 479 editing sites and 18 ones are in negative strand with 319 editing sites.

We ran MEME on such dataset by searching both palindromic and non-palindromic motifs with a length ranging from 6 to 50 nt. We bound the number of motifs to 50 palindromic and 50 non-palindromic.

From these 100 motifs we took only those with an *E*-value <0.05. Next, we filter out motifs that were contained in a set of human ultra-conserved sequences having no known editing site (Bejerano et al., 2004), with respect to DARNED and RADAR databases. Finally, a total of 16 motifs (4 palindromic and 12 non-palindromic) have been discovered.

In order to validate the filtered motifs, we performed a permutation test using 100 samples of 1,000 randomly taken 3′ UTR sequences (hg19) with masked repetitive regions. As shown in Table 1, only 13 motifs were significant (*p*-value <0.01).

**Table 1:** **Filtered motifs in ERs (47 edited regions)**.

Motif	Sequence (*Best possible match*)	Width	Type	*E*-value
1	CCAGGCTGGAGTGCAGTGGCGCAATCTCA	29	Non-palindromic	1E-126
2	GGATTACAGGCGTGAGCCACCGCGCCTGG	29	Non-palindromic	3,60E-123
3	GAGGTGCTGGGATTATAGGGG	21	Non-palindromic	8,50E-35
4	CCTGACCTCATGAGA	15	Non-palindromic	4,10E-22
5	AGACATGGAACCAACCTAAATGCCCACCA	29	Non-palindromic	9,40E-17
6	AGGAGGCAAAGGAAG	15	Non-palindromic	7,00E-11
7	TGGGATTGCAGGCAT	15	Non-palindromic	1,20E-06
8	TTTCATGGCTGCATAGTATTCTATTGTGT	29	Non-palindromic	1,00E-05
9	TGTAAATTAGTACAGCCTTTATGGAAAAC	29	Non-palindromic	2,90E-12
10	AGTCCCAGCTTCTCGAGAAGCTGGGACT	28	Palindromic	2,7E-97
11	TGCACCCCAGGCTGGGGTGCA	21	Palindromic	8,4E-50
12	CTTGTACTCCCAACATGTTGGGAGTACAAG	30	Palindromic	5,2E-72
13	CTTGAACCTCGGAGGTTCAAG	21	Palindromic	3,9E-28

## From Nucleotide Frequency to an Approach to Assessment of A-to-I RNA Editing Sites

Starting from the idea proposed by Pinto et al. (2014), we used a logistic regression technique to determine a model from which we can compute the probability that an adenosine in a non-repetitive region of the genome is affected by the A-to-I editing phenomenon. Our method, called AIRlINER, determines the editing probability of an adenosine by analyzing its flanking region of 10 nt. Such pattern is then combined with a similar model calculated from un-edited sequences, resulting in the estimation of an unbiased editing probability.

In order to train our method, we built a dataset composed of 30,280 sequences of 21 nt centered on an adenosine, from the human genome (hg19). According to their provenance, our dataset can be divided equally into two sets: known editing sites and random sites. For the purpose of retrieving known editing sites in non-repetitive regions, only human sites which do not have any repetitive elements in their flanking regions of 2,000 nt were selected from the RADAR database (Ramaswami and Li, 2014). Random sites were chosen by randomly selecting a number of sequences equal to that of the known editing sites. From such a selection, we excluded known editing sites in both repetitive and non-repetitive regions.

**Figure 2:**
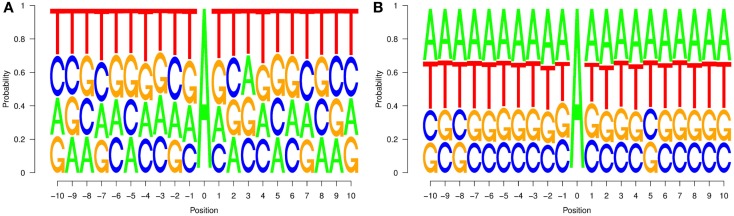
**Neighborhood preferences that we computed for experimentally verified editing sites in non-repetitive regions (A) and random sites (B) chosen among those for which no editing event is reported**. Neighborhood preferences are coherent with the upstream nucleotide distribution of editing site sequence contexts reported in Eggington et al. (2011).

From such a dataset, two probabilities *P*(*j*, *i*) and *P*’(*j*, *i*) can be computed: the first one corresponds to the probability of finding nucleotide *j* in position *i* of a region affected by editing, while the second one represents the probability of finding nucleotide *j* in position *i* of an un-ER. Starting from these probabilities, we computed the graphs in Figure 2, which represent the distributions of the nucleotides for the two types of regions.

Therefore, let *s* be a nucleotide sequence and *P*(*s*) its editing probability, using the previously defined probabilities we are able to train a logistic regression model such as:

$$\log\left(\frac{P\left(s\right)}{1-P\left(s\right)}\right)=\beta_{0}+\sum_{i=1}^{21}\beta_{i}P\left(s\left[i\right],i\right)-\sum_{i=1}^{21}\beta_{i}^{\prime }P^{\prime }\left(s\left[i\right],i\right),$$

where *s[i]* is the *i*-th nucleotide in a sequence. Now we can use this model to estimate the editing probability of any sequence of 21 nt centered on an adenosine, and if such probability is >0.5, we can say that such a sequence may be affected by editing.

To tune and validate our method, we applied a 10-fold cross validation procedure and computed a mean error. To compare our method with *InosinePredict*, we used a threshold to establish the presence or absence of editing in a specific sequence. Such a threshold was set to 9.6% for *InosinePredict*, as shown in Eggington et al. (2011). For our algorithm, we choose all sites for which an editing probability >0.5 is computed. We also took into account the fact that *InosinePredict* can produce predictions for both hADAR1 and hADAR2. We do not have this information in our dataset, so we chose to select the maximum score produced by *InosinePredict* for editing sites, and the minimum score for random sequences. Consequently, we are able to ensure a fair comparison with our method despite the absence of information on which ADAR affects each editing site.

In Tables 2 and 3, we show the confusion matrices computed using the previously described procedure. The two algorithms were applied to the dataset and the values computed for the central adenosines in each sequence were used to determine the presence or absence of editing. Our method significantly reduces the number of false negatives compared to *InosinePredict*, thus resulting in a better editing sites prediction quality. AIRlINER is also able to achieve a substantial reduction of false positives, even if nothing can be stated with certainty about them, as the absence of editing in these sites can also be determined by lack of experimental tests. The best quality in predicting editing sites, however, may reflect the fact that the random sequences classified as non-edited could be with high probability considered as such.

**Table 2:** **Confusion matrix computed by applying InosinePredict (Eggington et al., 2011) to our dataset**.

		Prediction outcome	
		Editing site	Non-editing site
Actual value	Editing sites	58.48	41.52
	Random sites	60.18	39.82

*Editing percentages for each sites have been divided into two classes (editing/non-editing) using the thresholds defined in Eggington et al. (2011)*.

**Table 3:** **Confusion matrix computed by applying AIRlINER to our dataset**.

		Prediction outcome	
		Editing site	Non-editing site
Actual value	Editing sites	71.18	28.82
	Random sites	34.05	65.95

*All editing sites for which editing probability is >0.5 were classified as editing while the remaining as non-editing*.

**Figure 3:**
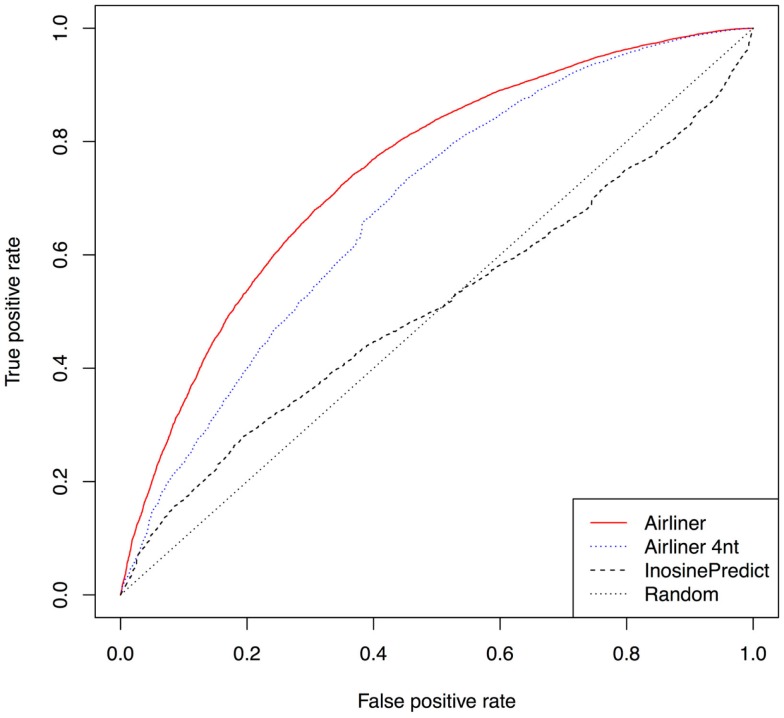
**Receiver operating characteristic curve (ROC) computed for the two prediction algorithms**. We also provide a ROC curve for a variant of our algorithm (AIRlINER 4 nt), which takes into account only the flanking region of 4 nt around an adenosine. Such a curve is useful to compare the performance with our algorithm using the same flanking region. AIRlINER shows an average area under the ROC curve (AUC) equal to 0.7466, while InosinePredict gets an AUC of 0.5072. AIRlINER 4 nt has an AUC of 0.7464.

Further confirmation of the quality of our methodology is represented by the receiver operating characteristic curves (ROCs), Figure 3, computed from the results produced by the two algorithms. The curves demonstrate a significant improvement in performance. Such curves also show that the threshold chosen to distinguish editing sites from non-editing ones does not affect the performance difference between the two algorithms. As a confirmation of this, *InosinePredict* obtains an average area under the ROC curve (AUC) of 0.5072, while AIRlINER reaches 0.7466. In Figure 3, we also compare a variant of our method, AIRlINER 4 nt, with *InosinePredict*. Such a variant computes the editing probability of an adenosine by considering its flanking region of 4 nt. This comparison shows that our strategy is superior to *InosinePredict* even when the prediction is calculated from this same region around an adenosine.

**Figure 4:**
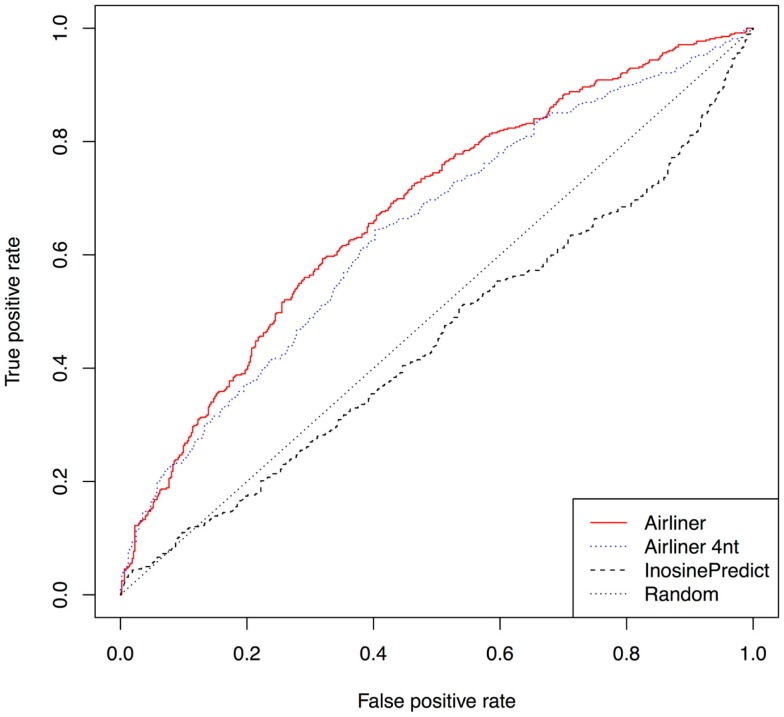
**Comparison between AIRlINER and InosinePredict by means of receiver operating characteristic curve (ROC) computed using the data set built from Bahn et al. (2012)**. Here we also show a ROC curve for a variant of the proposed algorithm (AIRlINER 4 nt), which takes into account only the flanking region of 4 nt around an adenosine. AIRlINER shows an average area under the ROC curve (AUC) equal to 0.6763, while InosinePredict gets an AUC of 0.4498. AIRlINER 4 nt has an AUC of 0.6435.

Furthermore, we investigated that ADAR acts on each editing site in our training set by building an additional data set from editing sites experimentally identified in (Bahn et al., 2012). Using human cell lines U87MG in which the gene expression of ADAR1 was repressed, the authors were able to identify about 4,000 ADAR1-specific editing sites. Four hundreds of such sites were identified in non-repetitive regions. From the latter, we have built a training set using the same procedure described above and trained our model. In Figure 4, we show the results of this experiment by means of ROC curves. Even in this case, the AIRlINER methodology is significantly better than *InosinePredict*. As further confirmation, we also computed the AUC, which amounts to 0.6763 for AIRlINER, and 0.4498 for *InosinePredict*.

Finally, to verify the quality of the editing sites predicted by our algorithm, we selected from the literature 52 experimentally validated sites by Sanger method and 7 sites validated as non-edited (as shown in Table S1 in Supplementary Material). We then applied the two methodologies and checked how many of them are correctly identified. AIRlINER is able to predict 42 of 52 editing sites and 5 of 7 non-editing sites while *InosinePredict* identifies 26 editing sites and 4 non-editing ones. More details can be found in the Table S1 in Supplementary Material.

AIRlINER is available as a web app at the following URL: http://alpha.dmi.unict.it/airliner/.

## Conclusion and Future Directions

RNA editing is a post-transcriptional phenomenon that occurs in eukaryotes and contributes to the diversity of transcriptome. A-to-I is the most common form of RNA editing in mammals, altering the sequence of primary RNA transcripts by adenosine deamination. In this last decade, computational methods and RNAseq based approaches to RNA editing discovery have emerged, contributing to the identification of more than a million editing events in human, many of which located close to or within Alu repeats. Despite the enormous efforts made so far, the biological significance of the editing phenomenon remains largely unknown.

In the first part of this work, we summarized some of the most important characteristics discovered for RNA editing. Inspired by literature, we investigated the presence of motifs in non-repetitive regions characterizing the editing events, finding a small set of candidates. Moreover, we considered the frequency of the 20 nt centered on each RNA editing site to compute the probability that an adenosine in a non-repetitive region of the genome may be affected by the A-to-I editing phenomenon. Our method, available on line, significantly reduces the number of false negatives with respect to existing methods, thus indicating a better editing-site prediction quality.

Future work will concern the use of different motif-detecting algorithms to confirm the consistency of our current findings. Motif detection methods may make use of information from the secondary structure of the editing regions with respect also to the different classes of ADAR. Finally, further investigation is needed to highlight any significant combination of motif patterns.

## Conflict of Interest Statement

The authors declare that the research was conducted in the absence of any commercial or financial relationships that could be construed as a potential conflict of interest.

## Supplementary Material

The Supplementary Material for this article can be found online at: http://www.frontiersin.org/Journal/10.3389/fbioe.2015.00018/abstract

## Footnotes

^1^http://darned.ucc.ie/

^2^http://rnaedit.com/
