# Supplementary material for: Knowledge in the Investigation of A-to-I RNA Editing Signals
Source: Front Bioeng Biotechnol. 2015 Feb 24;3:18. doi: 10.3389/fbioe.2015.00018 (PMC4338823; doi:10.3389/fbioe.2015.00018)
Supplement: Supplementary file 1 [file table_1.pdf]

Supplementary Material
Knowledge in the Investigation of A-to-I RNA editing signals

Giovanni Nigita[1,†], Salvatore Alaimo[2,†], Alfredo Ferro[3], Rosalba Giugno[3,$,*], Alfredo Pulvirenti[3,$,*]

[1]Department of Molecular Virology, Immunology and Medical Genetics, Ohio State University, Columbus, Ohio, USA

[2]Department of Mathematics and Computer Science, University of Catania, Catania, Italy

[3]Department of Clinical and Experimental Medicine, University of Catania, Catania, Italy

*** Correspondence:** Rosalba Giugno, Department of Clinical and Experimental Medicine, University of Catania, Via Santa Sofia, 78 | I-95122 Catania, Catania. Alfredo Pulvirenti, Department of Clinical and Experimental Medicine, University of Catania, Via Santa Sofia, 78 | I-95122 Catania, Catania.
giugno@dmi.unict.it; apulvirenti@dmi.unict.it

[†]Giovanni Nigita and Salvatore Alaimo have contributed equally to this work.

[$] Rosalba Giugno and Alfredo Pulvirenti have contributed equally to this work.

1. Supplementary Figures and Tables

1.1. Supplementary Tables

Supplementary Table 1. Comparison between AIRlINER and InosinePredict by means of experimentally validated A-to-I editing sites in literature. In the two score columns, boldface highlights sites predicted as editing, while italics identifies non-editing sites.The thresholds are 0.5 for AIRlINER and 9.6 for InosinePredict.

Genomic Position (hg19)	Chr	Strand	Gene	Type of region	Source	PubmedID	Reference	DNA Sequence	AIRlINER Score	InosinePredict Score
**EDITING**										
95160777	12	-	Unknown	O	Lymphoblastoid cell line	22327324	(Peng et al., 2012)	TCGATTAAGAAACATAAAGAT	*0,1948*	**17,2457**
102390869	12	+	Unknown	O	Lymphoblastoid cell line	22327324	(Peng et al., 2012)	CAGTAGAGACAGGGTTTCTTC	**0,5433**	*6,4933*
30717091	17	+	Unknown	O	Lymphoblastoid cell line	22327324	(Peng et al., 2012)	TACAAAAATTAGCTGGGCATG	*0,4830*	**100,0000**
114082694	X	+	5HT2C	EC	Cerebellum	19478186	(Li et al., 2009)	ACGTAATCCTATTGAGCATAG	**0,6739**	**34,5483**
277003	2	+	ACP1	EC	Spinal cord	22957051	(Picardi et al., 2012)	AAAAGTAATCAAGTTAAAACC	*0,0971*	*9,0628*
44153619	7	+	AEBP1	EC	Cerebellum	22912834	(Zhu et al., 2012)	GCTGGCTGGGAGGAGTCGGAG	**0,8000**	*1,9220*
23442122	14	-	AJUBA	E3	Lymphoblastoid cell line	23291724	(Ramaswami et al., 2013)	CTAAACCAAGATCAGTCAAAT	*0,2619*	*1,0935*
73980019	10	+	ANAPC16	I	Lymphoblastoid cell line	22327324	(Peng et al., 2012)	TCCGGGAGGCAGAGGTTGCTG	**0,6394**	*6,6744*
161035025	1	-	ARHGAP30	I	Lymphoblastoid cell line	22327324	(Peng et al., 2012)	GAGCCGGAGGATTTCACGACC	**0,7506**	*0,9674*
116937809	1	+	ATP1A1	EC	Brain	22957051	(Picardi et al., 2012)	GAATTTCCCTATCGATAATCT	**0,6185**	**34,8247**

Genomic Position (hg19)	Chr	Strand	Gene	Type of region	Source	PubmedID	Reference	DNA Sequence	AIRlINER Score	InosinePredict Score
**EDITING**										
103841636	8	-	AZIN1	EC	Cerebellum	22912834	(Zhu et al., 2012)	TCTTCTGTCGAAAGGTGTTAA	**0,5178**	*0,7447*
36147533	20	-	BLCAP	EC	Cerebellum	22912834	(Zhu et al., 2012)	CAACTCCCCGAACCCCTACTC	**0,7098**	*1,2228*
62458275	11	-	BSCL2	EC	Cerebellum	19478186	(Li et al., 2009)	GACTCCCTAGAAGTCCCGAGA	**0,5221**	*1,0823*
77979680	4	-	CCNI	EC	Breast Cancer	22912834	(Zhu et al., 2012)	CGATTTTTGGATAGGTTTTCT	**0,7096**	*0,6212*
9841186	12	+	CLEC2D	I	Lymphoblastoid cell line	22327324	(Peng et al., 2012)	ATGGTGGCATACGCCTGTAGT	**0,5945**	**40,6423**
126132620	9	+	CRB2	EC	Cerebellum	22912834	(Zhu et al., 2012)	CCCACCTGGTACCCATGGACC	**0,7307**	**52,7592**
156736808	5	+	CYFIP2	EC	Cerebellum	19478186	(Li et al., 2009)	CAGATACATTAAGACCAGTGC	**0,5193**	**100,0000**
32456380	9	-	DDX58	E3	Brain	23291724	(Ramaswami et al., 2013)	CATCATTCGTATTTTTACCGA	**0,7275**	**32,6700**
179044110	1	+	FAM20B	E3	Lymphoblastoid cell line	22327324	(Peng et al., 2012)	AGATCACCTGAGGCCAGGAGT	**0,6211**	*3,6274*
55765797	14	+	FBXO34	I	Lymphoblastoid cell line	22327324	(Peng et al., 2012)	GTTTGGAGAAAAGTCTGACGA	*0,3973*	**20,0042**
130629624	7	-	FLJ43663	E3	Brain	23291724	(Ramaswami et al., 2013)	GAGTAGAAGGAAATTAAGAAT	*0,1817*	*0,9987*
153579950	X	-	FLNA	EC	Brain	19478186	(Li et al., 2009)	CACGGAGTGGACTTCCGATCT	**0,6034**	*0,8778*
58141801	3	+	FLNB	EC	Cerebellum	22912834	(Zhu et al., 2012)	ATGAGCCTTCAGGTGAGATGC	**0,5081**	**14,4591**
151358319	X	-	GABRA3	EC	Cerebellum	19478186	(Li et al., 2009)	TTGTCTGCCGATACTTGGTCA	**0,7825**	*0,6529*
57864624	12	+	CLI1	EC	Cerebellum	22912834	(Zhu et al., 2012)	CATGGATGCTAGAGGGCTACA	**0,7451**	**68,7209**
158257875	4	+	GRIA2	EC	Cerebellum	19478186	(Li et al., 2009)	GCCTTTATGCAGCAAGGATGC	**0,5342**	*4,5598*
122598962	X	+	GRIA3	EC	Cerebellum	19478186	(Li et al., 2009)	CTCAGCATTAAGGTGGGTGGA	**0,6628**	**37,6124**
105804694	11	+	GIRA4	EC	Brain	21984433	(Silberberg et al., 2012)	TTCCTCATTAAGGTGGGTGGA	**0,6766**	**37,6124**
30953750	21	-	GRIK1	EC	Cerebellum	19478186	(Li et al., 2009)	ACATGGAACGACGTACTCTCG	**0,5199**	*1,2055*
102337702	6	+	GRIK2	EC	Cerebellum	19478186	(Li et al., 2009)	CTGCTGGCTTACTTGGGTGTC	**0,8491**	**83,5633**
57976286	4	-	IGFBP7	EC	Cerebellum	19478186	(Li et al., 2009)	GTCATGGGGGACGGCCGCGGC	**0,8084**	*1,1401*
44181739	12	+	IRAK4	E3	Lymphoblastoid cell line	22327324	(Peng et al., 2012)	GGACACCTGTAGTCCCAGCTA	**0,7060**	**57,0387**
5021742	12	+	KCNA1	EC	Cerebellum	19478186	(Li et al., 2009)	TGTGCTAACAATTGCCCTGCC	**0,7873**	**14,0327**
79397298	10	-	KCNMA1	EC	Cerebellum	22912834	(Zhu et al., 2012)	CGCAGATCCGACTCTACCAAG	**0,5974**	*1,1990*
73843194	6	+	KCNQ5	EC	Cerebellum	22912834	(Zhu et al., 2012)	CCCAGGGGCCAGAGTATTAAG	**0,5789**	*7,1646*
241700776	2	-	KIF1A	EC	Cerebellum	22912834	(Zhu et al., 2012)	GTCGAGTGTGAGGGCGAGACA	**0,7037**	*3,4524*
28662186	1	+	MED18	E3	Lymphoblastoid cell line	22327324	(Peng et al., 2012)	TACAAAATTTAGCCGGGCATG	**0,5502**	**62,5820**
417757	16	-	MRPL28	EC	Spinal cord	22957051	(Picardi et al., 2012)	GAGGCGGTGTATCTAGAACTT	**0,7056**	**22,3593**
15986074	1	-	NCSTN	EC	Cerebellum	22912834	(Zhu et al., 2012)	TAATACACTTACGTATAATTA	*0,1563*	**69,4973**
75646086	15	+	NEIL1	EC	Cerebellum	19478186	(Li et al., 2009)	ACAGGGGGCAAAGGCTACGGG	**0,6865**	**21,1966**
25987370	14	+	NOVA1	EC	Brain	22957051	(Picardi et al., 2012)	AGCTAATTGGATTGGATGACC	**0,6428**	*0,6752*

Genomic Position (hg19)	Chr	Strand	Gene	Type of region	Source	PubmedID	Reference	DNA Sequence	AIRlINER Score	InosinePredict Score
**EDITING**										
8173299	17	+	PFAS	E3	Lymphoblastoid cell line	22327324	(Peng et al., 2012)	GGGTTCAAGCAATTTTCCTGC	**0,5757**	*5,2469*
45567409	12	-	PLEKHA9	EC	Lymphoblastoid cell line	22327324	(Peng et al., 2012)	GAATTATAAAAGTTGTTCTAG	*0,3064*	**51,4919**
157333469	7	-	PTPRN2	EC	Cerebellum	22912834	(Zhu et al., 2012)	GTCGCGCTTGAGTTTGACGAG	**0,7717**	*2,8627*
34923319	21	+	SON-1	EC	Lymphoblastoid cell line	22327324	(Peng et al., 2012)	CTGGGGCACTAGAGTTGCCTG	**0,8611**	**77,1102**
29681719	16	+	SPN	E3	Lymphoblastoid cell line	22327324	(Peng et al., 2012)	CTGAGGCAGAAGAATTGCTTG	**0,5713**	**41,4219**
225974614	1	+	SRP9	EC	U87MG	21725310	(Ju et al., 2011)	GCATGATCATAGCTCACTGCA	**0,7247**	**67,5020**
44120349	6	+	TMEM63B	EC	Astrocytoma cell lines overexpressing ADAR2	22957051	(Picardi et al., 2012)	TACGAGTTCCAGTTTGGCGCA	**0,7220**	*6,5476*
54955567	X	+	TRO	EC	Cerebellum	22912834	(Zhu et al., 2012)	CACCAGCACTAGTTTCAGCAG	**0,7445**	**90,1242**
9876560	3	+	TTLL3	EC	Cerebellum	22912834	(Zhu et al., 2012)	CTTGATTTCAAGGTGGCACCC	**0,7084**	**28,0769**
52604985	13	+	UTP14C	EC	Lymphoblastoid cell line	22327324	(Peng et al., 2012)	CATCAGGTACAAGTGCTTCCA	*0,4326*	*4,2981*
37037181	19	-	ZNF529	E3	Lymphoblastoid cell line	23291724	(Ramaswami et al., 2013)	TATATTGGTCAAGGATACATA	*0,1506*	**11,0583**
**NON EDITING**										
89160357	2	-	Unknown	O	Lymphoblastoid cell line	22327324	(Peng et al., 2012)	GCGAGGTTTAAATTTTGGAAT	*0,4962*	**34,0340**
95160789	12	-	Unknown	O	Lymphoblastoid cell line	22327324	(Peng et al., 2012)	CATAAAGATCATCTCTACCCC	*0,4478*	*7,6514*
32456371	9	-	DDX58	I	Lymphoblastoid cell line	23291724	(Ramaswami et al., 2013)	GTCGAAAATCATCATTCGTAT	*0,3480*	*5,3436*
28662180	1	+	MED18	E3	Lymphoblastoid cell line	22327324	(Peng et al., 2012)	GAAAAATACAAAATTTAGCCG	*0,2198*	**26,8839**
234775041	2	-	MSL3L2	EC	Lymphoblastoid cell line	22327324	(Peng et al., 2012)	TTAACAGTCCATGCTCGAAGG	*0,4911*	*2,4136*
54946784	19	+	TTYH1	EC	Brain	22957051	(Picardi et al., 2012)	TCTTCCTGCTACTCTTCTCCC	**0,8735**	**47,8726**
8578035	19	-	ZNF414	EC	Unknown	22957051	(Picardi et al., 2012)	CCTCGGGACGACGTAGGGAGG	**0,7098**	*1,4771*

References

Ju, Y. S., Kim, J.-I., Kim, S., Hong, D., Park, H., Shin, J.-Y., et al. (2011) . Extensive genomic and transcriptional diversity identified through massively parallel DNA and RNA sequencing of eighteen Korean individuals. Nature Genetics, 43(8), 745–752. doi:10.1038/ng.872

Li, J. B., Levanon, E. Y., Yoon, J.-K., Aach, J., Xie, B., Leproust, E., et al. (2009) . Genome-wide identification of human RNA editing sites by parallel DNA capturing and sequencing. *Science* , *324* (5931), 1210–1213. doi:10.1126/science.1170995

Peng, Z., Cheng, Y., Tan, B. C.-M., Kang, L., Tian, Z., Zhu, Y., et al. (2012) . Comprehensive analysis of RNA-Seq data reveals extensive RNA editing in a human transcriptome. *Nature Biotechnology* , *30* (3), 253–260. doi:10.1038/nbt.2122

Picardi, E., Gallo, A., Galeano, F., Tomaselli, S., & Pesole, G. (2012) . A novel computational strategy to identify A-to-I RNA editing sites by RNA-Seq data: de novo detection in human spinal cord tissue. PLoS ONE, 7(9), e44184. doi:10.1371/journal.pone.0044184.s005

Ramaswami, G., Zhang, R., Piskol, R., Keegan, L. P., Deng, P., O'Connell, M. A. A., & Li, J. B. (2013) . Identifying RNA editing sites using RNA sequencing data alone. *Nature Methods* , *10* (2), 128–132. doi:10.1038/nmeth.2330

Silberberg, G., Lundin, D., Navon, R., & O ̈hman, M. (2012). Deregulation of the A-to-I RNA editing mechanism in psychiatric disorders. Human Molecular Genetics, 21(2), 311–321. doi:10.1093/hmg/ddr461

Zhu, H., Urban, D. J., Blashka, J., McPheeters, M. T., Kroeze, W. K., Mieczkowski, P., et al. (2012) . Quantitative Analysis of Focused A-To-I RNA Editing Sites by Ultra-High-Throughput Sequencing in Psychiatric Disorders. PLoS ONE, 7(8), e43227. doi:10.1371/journal.pone.0043227.s015
